# Swahili translation and validation of the Warwick Edinburgh Mental Wellbeing Scale (WEMWBS) in adolescents and adults taking part in the girls’ education challenge fund project in Tanzania

**DOI:** 10.1186/s12955-023-02119-9

**Published:** 2023-05-10

**Authors:** Oyinlola Oyebode, Manuel Torres-Sahli, Deus Kapinga, Elizabeth Bruno-McClung, Rebecca Willans, Neha Shah, Lydia Wilbard, Louise Banham, Sarah Stewart-Brown

**Affiliations:** 1 Warwick Medical School, University of Warwick, Coventry, UK; 2 Wolfson Institute of Population Health, Queen Mary, University of London, London, United Kingdom; 3 School of Social Sciences and Humanities, University of Loughborough, Loughborough, UK; 4 CAMFED Campaign for Female Education, Dar Es Salaam, Tanzania; 5 Centre for Mental Health, City University, London, UK; 6 Foreign, Commonwealth and Development Office, London, United Kingdom

**Keywords:** Mental wellbeing, Adolescents, Translation, Measurement, Confirmatory factor analysis, Tanzania, Education

## Abstract

**Background:**

The Warwick Edinburgh Mental Wellbeing Scale (WEMWBS) is validated for measuring mental wellbeing in populations aged 11 + and has been translated into 30 + languages. The aims of this study were a) to translate and validate WEMWBS for use in Swahili-speaking populations to facilitate measurement and understanding of wellbeing, evaluation of policy and practice, and enable international comparisons; and b) to examine sociodemographic characteristics associated with higher and lower mental wellbeing in participants in the Girls’ Education Challenge (GEC) project in Tanzania.

**Methods:**

A short questionnaire including WEMWBS and similar scales for comparison, socio-demographic information, and self-reported health was translated into Swahili using gold standard methodology. This questionnaire was used to collect data from secondary school students, learner guides, teacher mentors and teachers taking part in the GEC project in Tanzania. Focus groups were used to assess acceptability and comprehensibility of WEMWBS and conceptual understanding of mental wellbeing. These were audio-taped, transcribed and analysed thematically. Internal consistency of WEMWBS, correlation with comparator scales and confirmatory factor analysis were completed as quantitative validation. Finally, multivariable logistic regression was used to explore associations between individual characteristics and ‘high’ and ‘low’ mental wellbeing, defined as the highest and lowest quartile of WEMWBS scores.

**Results:**

3052 students and 574 adults were recruited into the study. Participants reported that WEMWBS was understandable and relevant to their lives. Both WEMWBS and its short form met quantitative standards of reliability and validity, were correlated with comparator scales and met the criteria to determine a single factor structure. For students in the GEC supported government schools: mental wellbeing was higher in students in the final two ‘forms’ of school compared with the first two. In addition: being male, urban residence, the absence of markers of social marginality and better self-reported health were all significantly associated with better mental wellbeing. For adults, urban residence and better self-reported health were associated with better mental wellbeing.

**Conclusions:**

The Swahili translation of WEMWBS is available for use. Further work to explore how to intervene to increase mental wellbeing in vulnerable GEC participants is needed.

**Supplementary Information:**

The online version contains supplementary material available at 10.1186/s12955-023-02119-9.

## Background

Understanding and measuring wellbeing is a policy priority. In addition to its very important intrinsic value, wellbeing has instrumental value because it drives population health as well as associated health care costs, and social and economic progress, with impacts on employment, productivity, criminal activity, prosocial behaviours and education [1].

The Warwick Edinburgh Mental Wellbeing Scale (WEMWBS) is validated for measuring mental wellbeing in populations aged 13 and above in the UK [2], with the short-form validated in populations aged 11 and above [3]. It has been translated into more than 30 languages, and many translations and validations have been published [4]. It is collected and reported as part of UK national statistics and has been used extensively to evaluate interventions [5–7].

The primary aim of this study was to translate and validate WEMWBS and its short form for use in adolescent and adult Swahili-speaking populations. This will facilitate measurement and understanding of wellbeing in Swahili speaking populations, evaluation of policy and practice where the target populations are Swahili speaking or include Swahili speaking people, and enable international comparisons. The secondary aim of this study was to describe mental wellbeing in the participants of the Tanzanian Girls’ Education Challenge (GEC) fund project, run by the Campaign for Female Education (CAMFED) and funded through the UK’s Foreign, Commonwealth and Development Office. Specifically, we aimed to examine sociodemographic characteristics associated with higher and lower wellbeing in this population.

## Methods

This was a mixed methods study including qualitative and quantitative data collection to support the primary aim of the study, and quantitative data collection to answer the secondary aim of the study.

### Translation

Translations followed gold standard methods. Firstly, two translators, both with Swahili as their mother-tongue, independently translated WEMWBS from English into Swahili. Secondly, the translators shared their independent translations and worked together to produce one common consensus version of WEMWBS in Swahili. Notes were taken to document issues addressed and how they were resolved. The Swahili version of WEMWBS was sent to two translators who had never seen the original WEMWBS and they independently translated the Swahili version back into English. Although ideally, these would have been people with English as their mother-tongue, our translators were native Swahili speakers with fluent English. The two new English versions and associated notes were sent to Sarah-Stewart Brown (SSB) (one of the WEMWBS developers) who compared these against the original WEMWBS with any minor points for further discussion noted at this point. Further discussion with a native Swahili speaker (not one of the previous translators), alongside the translations and notes, further refined the translated version into the final Swahili WEMWBS.

### Sample size, setting and participants

There is a lack of consensus about how to calculate sample size for validation studies for scales, with recommendations varying from a participant to item ratio of between 2 and 20 participants per item, to absolute recommendations e.g.; Comrey and Lee state 100 = poor, 200 = fair, 300 = good, 500 = very good, ≥ 1000 = excellent [8]. We considered both the sample required for the validation study, as well as our secondary aim (to investigate the determinants and distribution of wellbeing in the population examined). In order to sample enough adults to be able to have an appropriate sample size for the validation in adults, we planned to visit 100 schools, and for this reason we were able to collect data from far more students that necessary for the validation studies alone at little extra expense. These data allowed us to examine the associations between the WEMWBS and socio-demographic characteristics of participants.

CAMFED Tanzania operates within 28 districts (15 rural and 13 peri-urban districts); we purposively selected 5 of these districts based on criteria to allow a cross section of district characteristics (rural, peri-urban; coastal, inland; higher and lower scores on national exams) and bearing in mind practical considerations (for example, journey times for fieldworkers, budgetary constraints). We drew up lists of GEC schools in each of the 5 selected districts and randomly selected schools in each for our sample according to the number of supported schools in the district (between 10–34 selected schools per district = 90 schools in total).

At each school a target sample of 34 students were randomly selected from the school student roll (in a few schools there were fewer than 34 students in the specified forms), additionally 2 teachers were randomly selected from the staff list. Each school has a single teacher mentor and usually 3 learner guides engaged in delivering the GEC fund project all of whom were invited to take part in the study. These approximately 40 individuals per school were invited to participate. The fieldworker visited the school and described the project, giving the opportunity for prospective participants to ask questions, and provided the prospective participants with participant information sheets and consent forms (including second copies of these for parents and guardians if the participant was a student). They were given a minimum of 24 h to consider the information and return the consent form. No student or teacher declined consent although we planned to randomly select a further participant from the appropriate group if that situation arose (including a teacher to replace a teacher mentor in the situation that a teacher mentor declined participation).

Eight schools were purposively chosen for focus groups with students, and either older (forms 3 and 4- usually aged 17 +) or younger (forms 1 and 2- usually aged 15 +) adolescents from that school were invited to participate (four focus groups of younger or two of older adolescents respectively). Eight different schools were purposively chosen for focus groups with adults and all teachers and learner guides taking part in quantitative data collection at those schools were invited to participate. For the adult focus groups, teachers or learner guides came together from across participating schools for the four focus groups. The schools were chosen in order to reflect varying school characteristics. Equal numbers of urban and rural schools participated in qualitative data collection.

### Data collection

A questionnaire to collect quantitative data was designed by the research team comprising OO, SSB, LB, DK and LW, based on questions that have been used in existing established survey questionnaires. It was designed to take approximately 15 min to complete.

The quantitative data set included: the WEMWBS, comparator questions (The World Health Organisation- Five Well-being Index (WHO5), the 12-item General Health Questionnaire (GHQ-12), Office for National Statistics-4 (ONS-4) and self-reported health), socio-demographic variables (age, sex, ‘marginality indicators’ (a set of 20 questions based on Tanzania’s national guidelines for the Care and Support of the Most Vulnerable Children)) and questions relating to GEC exposure, collected directly from participants via questionnaires on tablets using the Open Data Kit application. Please note that the short form of WEMWBS (SWEMWBS) uses 7 of the 14 WEMWBS items.

Data collection was completed by a team of 20 fieldworkers from the Tanzanian chapter of CAMA, the pan-African network of educated young African women (graduates of the CAMFED programmes), after training by the CAMFED Monitoring and Evaluation team and OO, and with continuous supervision by DK, the Head of Monitoring and Evaluation at CAMFED.

At the end of every day of data collection, the data were submitted into the CAMFED server for secure storage. These data were checked for quality, specifically that the data matches what is expected (the no. of participants for example, and that there were no duplicates).

Qualitative data were collected in 12 focus groups run after quantitative data collection in a subset of the schools, as part of the validation, in order to ask about participants’ experiences of completing the questionnaire, particularly their thoughts on the different wellbeing questions asked. In addition, the topic guide included questions on the concept of mental wellbeing more generally. Focus groups were recorded in Swahili on an encrypted audio recording device then transcribed by the CAMA fieldworkers who collected the data. Data were anonymised during transcription. Translation was then completed by CAMA fieldworkers and some English teachers based within GEC secondary schools, known to CAMFED and previously commissioned for similar work. Twelve focus groups were used, based on pragmatic considerations and with the expectation that data saturation would be reached (it was).

### Qualitative analysis

Both inductive and deductive qualitative analytical techniques were used to analyse the content of the transcripts in English. This analysis was part of the validation and explored the acceptability and comprehensibility of the WEMWBS tools (and comparator tools) as well as what the concept of wellbeing means to the participants and whether WEMWBS captured this (with the deductive analysis specifically testing whether concepts relating to WEMWBS were present in the testimony). Coding was completed by one researcher (BW) and with reflection with a second researcher throughout the process (OO).

### Confirmatory factor analysis

Confirmatory factor analysis (CFA) was performed for validation of the Swahili (S)WEMWBS in three steps: (1) specification of theoretical model, (2) modification based on potentially misspecified parameters, and (3) assessment of global fit of the modified model and the relative difference (RD) of parameters between theoretical and the modified model ($\frac{{\theta }_{modified}-{\theta }_{theoretical}}{{\theta }_{modified}}$). The modified model was considered appropriate when (i) no parameters were severely misspecified, (ii) RD in loadings compared to the theoretical model was negligible (max. RD < 10%), and (iii) global fit indexes fell within pre-defined ranges of acceptability. We used thresholds for good fit (CFI & TLI > 0.95, RMSEA & SRMR < 0.06) and acceptable fit (Comparative Fit Index (CFI) & Tucker-Lewis Index (TLI) > 0.90, Root Mean Square Error of Approximation (RMSEA) & Standardised Root Mean Square Residual (SRMR) < 0.08). This three-step CFA allowed us to model sources of error that could be substantively irrelevant (i.e., produced negligible parameter bias or RD) but improved the precision of reliability indices (diminishing the risk of overestimating scores' reliability).

We used MacDonald's ω as a reliability index because it accounts for factor structure (including, for example, correlated residuals) and is more appropriate when the loadings vary – α is reported for discussion. Two sets of cut-off values have been considered for ω. The first is a traditional, scaled set of cut-off values: excellent (> 0.90), good (> 0.80), acceptable (> 0.70), questionable (> 0.60) and poor (> 0.50). The second is practical: adequate for general purpose or research > 0.70, best for high-stakes decisions > 0.90). We also evaluated factor score determinacy (FD: acceptable > 0.80, good > 0.90 – Grice, 2001) for use of loading-based scores and construct replicability (H: acceptable > 0.70, good > 0.85 – Hancock & Mueller [9]) or specifying SEM.

Measurement invariance was examined following Wu and Estabrook’s [10] recommendations for models with ordinal indicators (such as WEMWBS), assessing changes in the same fit indices used for global fit evaluation. Following Rutkowski and Svetina [11] changes in CFI of up to -0.02 and RMSEA of up to 0.03 were considered appropriate for tests of metric/weak invariance, while ΔCFI ≥ -0.01 for and ΔRMSEA ≤ 0.01 were considered appropriate for scalar/strong invariance tests. We also considered Chen's [12] recommendation of a change in SRMR ≤ 0.030 (for metric invariance) or ≤ 0.015 (for scalar or residual invariance).

The data for indicators in all instruments was skewed due to increased concentration in the higher (better) values; with endorsement of the highest response category over 40% in some items. All instruments presented sparse data with more than half of their items having < 0.5% of endorsement in the lowest (i.e., worse scoring) response category. Therefore, following DiStefano et al. [13] guidelines, we evaluated each model both with and without collapsed response categories. Models were fitted using unweighted least squares estimator with mean and variance adjusted (scale-shifted approach, ULSMV) since is best suited for ordered data with skewed distributions.

Details and justification about cut-off values, specification of the models, assessment, and modification of misspecified parameters, and chosen estimator are given in Supplemental Materials.

### Multivariable analysis

To address the secondary aim of this study, we defined groups within the population, those with low mental wellbeing, scoring below the lowest quartile in our data; those with high mental wellbeing scoring above the highest quartile. Logistic regression models were used to generate odds of low mental wellbeing compared to the rest of the population and to generate the odds of high mental wellbeing compared to the rest of the population in two sets of models. Unadjusted models included each characteristic in turn (gender, age group, location (urban vs rural), self-reported health, form (adolescents only), marginality (adolescents only), education (adults only), role (adults only), main occupation (adults only)) and fully adjusted multivariable models included a subset of characteristics of interest, selected based on theory to reduce multicollinearity (sex, location, self-reported health, form(adolescents only), marginality (adolescents only), role (adults only) and education (adults only)).

### Software

CFA and the correlation among test scores were modelled within the R language and environment (r core team, 2020) using the *lavaan* package [14] and related helper functions within the *semTools* package [15] such as *reliability* (for α and ω) and *miPowerFit* (for EPC and MI analysis), as well as the *BifactorIndicesCalculator* [16] for H and FD indices. Qualitative analysis was completed using Microsoft Word. Descriptive statistics, univariable and multivariable regression analyses were conducted in StataIC version 16.1.

## Results

3052 students and 574 adults were recruited into the study and provided data to the quantitative analyses. The characteristics of the included participants are shown in Table 1 (students) and Table 2 (adults). The 96 qualitative participants were a subset of these (32 adults in 4 focus groups: 16 learner guides, 16 teachers; and 64 students in 8 focus groups: 32 aged 15–16, 32 aged 17 +).

**Table 1:** Characteristics of Student Survey Participants

Characteristics	*n* (%)
**Gender**	
Female	1588 (52.03)
Male	1458 (47.77)
Other	6 (0.20)
**Age group**	
15 years	1099 (36.01)
16 years	881 (28.87)
17 years	681 (22.31)
18 years	291 (9.53)
19 + years	100 (3.28)
**Form**	
Form one	759 (24.87)
Form two	974 (31.91)
Form three	834 (27.33)
Form four	485 (15.89)
**Location**	
Rural	2374 (77.79)
Urban	678 (22.21)
**Marginality Indicator**	
Not Marginalized	210 (6.88)
Marginalized	2842 (93.12)
**Marginality Score**	
0	210 (6.88)
1	363 (11.89)
2	394 (12.91)
3	429 (14.06)
4	348 (11.40)
5	332 (10.88)
6	302 (9.90)
7	219 (7.18)
8	184 (6.03)
9	99 (3.24)
10	83 (2.72)
11 +	89 (2.92)
**Self-Reported Health**	
Excellent	683 (22.38)
Very good	247 (8.09)
Good	1250 (40.96)
Fair	835 (27.36)
Poor	37 (1.21)

**Table 2:** Characteristics of Adult Survey Participants

Characteristics	*n* (%)
Gender	
Female	459 (79.97)
Male	115 (20.03)
Age group	
15–19 years	73 (12.72)
20–24 years	162 (28.22)
25–29 years	75 (13.07)
30–34 years	160 (27.87)
35–39 years	79 (13.76)
40 + years	25 (4.36)
Role	
Learner Guide	304 (52.96)
Teacher Mentor	90 (15.68)
Teacher	180 (31.36)
Location	
Rural	443 (77.18)
Urban	131 (22.82)
Education	
No Higher Education	286 (49.83)
Some Higher Education	288 (50.17)
Self-Reported Health	
Excellent	185 (32.23)
Very good	87 (15.16)
Good	264 (45.99)
Fair to Poor	38 (6.62)
Main Occupation	
Teacher	266 (46.34)
Professional	26 (4.53)
Skilled Non Manu	100 (17.42)
Skilled Manual	111 (19.34)
Unskilled	10 (1.74)
Other	61 (10.63)

### Qualitative validation

Overall WEMWBS was understood by the participants, and participants felt the way they answered would give someone a good indication of their wellbeing. The participants reported finding it easy to complete and answering honestly with frequent use of words such as “self-explanatory” and commenting on the relevance of the questions to their lives.

Learner-guide: I was free and I answered honestly from the heart because it is something I deal with every day

Some specific WEMWBS items (confidence, feeling cheerful, dealing with problems well and feeling relaxed) were directly referenced when participants discussed what they considered to be important indicators of good wellbeing.

Leaner-guide: If you are in good wellbeing you will have peace and you will also be happy and enjoy your life.

Participants also presented the idea that mental wellbeing includes making a positive contribution to society, and considered that personal wellbeing might be related to community wellbeing. One student also talked about obedience, which might be considered a way of relating positively to the community.

Teacher: Good wellbeing means a lot to me and to someone else. First I grow confident, I have good relationships with other people, thirdly it brings me positive development, because I grow up confident, I am healthy because good wellbeing plays a big part in the health and life of the community around you, so I see it leads to development even in your community.

Student: Good wellbeing is the one that leads a person to do something that he has agreed to for the whole community

Student: Wellbeing is being disciplined and obedient all the time.

When asked to describe poor mental wellbeing participants across the focus groups suggested criteria relating to problem behaviour and its consequences, as well as negative feelings and perspectives; some responses included:

Student: He can also be a person all the time knowing that citizens hate him because of the behaviour he goes with which is not good, he can also be lonely due to his own situation.

Student: He can also have negative thoughts and decide to do something wrong. Sometimes he can be harsh eg he can rob, or hurt others.

Student: actually it’s not easy to identify them but through different researchers these people have pain, feels lonely and abandoned. Secondly these people don’t trust themselves when they’re in front of people also feel unloved and separate themselves from people, also they feel scared and don’t trust themselves.”

Student “sometimes they can use marijuana”.

Teacher "most of the time he will do things that are against the school and the community for instance if a person is a drunkard or a thief in that community they will not like him.”

All groups liked being asked positively framed questions and several commented on not liking the negatively framed questions included in the survey. Certainly the testimony indicated that WEMWBS was understood, generally applicable to the participants’ lives and captured the main elements of mental wellbeing as they described it. There were no obvious differences in conceptualisation of mental wellbeing between the students and the adults who participated.

### Quantitative validation

#### Sparse data and collapsed response categories

The dataset presented sparse data for WEMWBS, i.e., response categories with < 10% endorsement in several items. Because this can produce significant differences in parameter estimates, standard errors, chi-square-values, and chi-square-based fit indices, we calculated CFA models with both sparse and collapsed data (merging low-endorsement response categories with adjacent values) before assessing their psychometric qualities. Using collapsed response categories in the presence of sparse data has been shown to improve the precision of parameter and standard errors with ULSMV estimator and is a better option than using robust maximum likelihood estimators while treating the data as interval numeric [13]. Since the parameter estimates with collapsed data presented low RD (only three items among all instruments with RD > 10%), we reported the results with non-collapsed data to avoid overestimating fit or reliability indices. In particular, (S)WEMWBS models presented negligible parameter RD when response categories were collapsed (max RD = 2.76%).

Only for measurement invariance did we use collapsed versions of WEMWBS' items 1, 6, 7, and 12. We merged the lowest (worst) response category with the second-lowest because at least one group (e.g., adults, males) presented zero observations in the lowest one.

#### (S)WEMWBS CFA

The theoretical model for WEMWBS (unidimensional, no correlated residuals) presented acceptable global fit indices (Table 3). Following the analysis of MI and EPC, we included three correlated residuals in the model: items 1–2 (first two items of the instrument), 4–13 (both about feeling "interested"), and items 8–14 (concerned with feeling "good"/"cheerful"). This model presented optimal global fit indices. The RD of factorial loads between the theoretical and modified models was negligible (mean = 2.1%, max = 4.9%) — therefore indicating that the correlations of residuals were substantively irrelevant, affecting the reliability of the scale but not the meaning of the measured construct.

**Table 3:** Global fit and reliability indices for theoretical, modified and collapsed-category models of (S)WEMWBS

Model	Global goodness-of-fit								Reliability,			
	X^2^	*df*	X^2^ / *df*	*p-value*	CFI	TLI	RMSEA(CI)	SRMR	α	ω	H	FD
*WEMWBS*												
Theoretical	1662	77	21.5	< .0001	.929	.916	.075 (.072-.078)	.051	.88	.74	.88	.94
Correlated Residuals	1085	73	15.1	< .0001	.953	.943	.062 (.059-.066)	.042	.88	.71	.88	.94
Collapsed categories	982	74	13.3	< .0001	.959	.950	.058 (.055-.061)	.042	.88	.71	.89	.94
*SWEMWBS*												
Theoretical	271	14	19.4	< .0001	.966	.949	.071 (.064-.079)	.039	.79	.73	.79	.89
Correlated Residuals	153	13	11.8	< .0001	.981	.970	.055 (.047-.062)	.029	.79	.70	.79	.89
Collapsed categories	129	13	9.9	< .0001	.984	.974	.050 (.042-.058)	.028	.79	.70	.79	.89

*CFI* Comparative Fit Index. *TLI* Tucker-Lewis Index. *RMSEA* Root Mean Square Error of Approximation. *SRMR* Standardized Root Mean Square Residual. The models with collapsed categories were analysed with the two lowest (worst scoring) categories merged into one, including the same modifications as the models with correlated residuals

For SWEMWBS’s scores, where the initial model showed an optimal fit except for RMSEA. As with WEMWBS, correlation of residuals for items 1–2 was modelled, resulting in acceptable RD in loading parameters (mean = 3.6%, max = 9.0%) and optimal global fit indices (Table 3).

Based on the models with correlated residuals, unit-weighted composite scores for WEMWBS and SWEMWBS presented practically equal and acceptable levels of reliability (ω = 0.71 and 0.70). Nonetheless, the factor scores for WEMWBS offered higher determinacy (FD = 0.94 *vs* 0.89) and replicability (H = 0.88 *vs* 0.79), positioning it in the optimal range for both (FD ≥ 0.90, H ≥ 0.85) while SWEMWBS fell in the acceptable range (FD ≥ 0.80, H ≥ 0.70).

Factorial loads were mostly good (≥ 0.50) for both sets of items with only item 4 falling significantly below 0.50 (0.35; 95%CI = 0.32–0.38) in the WEMWBS and item 13 being also < 0.50, though not significantly so (0.48; 95%CI = 0.45–0.51) (Table 4). Both items are not present in the short WEMWBS. WEMWBS and SWEMWBS appeared to measure the same construct since the RDs of the loadings of the common items were low (mean = 3.6%, max = 9.0%).

**Table 4:** Factorial loadings for theoretical, modified and collapsed-category models of (S)WEMWBS

Item	WEMWBS			SWEMWBS		
	Original	Corr. Errors	Coll	Original	Corr. Errors	Coll
1.I’ve been feeling optimistic about the future	.53 (.51-.56)	.51 (.48-.54)	.51 (.48-.54)	.57 (.54-.60)	.53 (.49-.56)	.52 (.49-.56)
2.I’ve been feeling useful	.57 (.54-.59)	.55 (.52-.57)	.54 (.52-.57)	.61 (.58-.64)	.56 (.54-.59)	.56 (.53-.59)
3.I’ve been feeling relaxed	.56 (.53-.59)	.56 (.54-.59)	.57 (.54-.60)	.55 (.52-.58)	.56 (.53-.59)	.56 (.53-.59)
4.I’ve been feeling interested in other people	.37 (.34-.40)	.35 (.32-.38)	.35 (.32-.38)			
5.I’ve had energy to spare	.65 (.63-.67)	.65 (.63-.68)	.67 (.64-.69)			
6.I’ve been dealing with problems well	.62 (.59-.64)	.62 (.60-.65)	.64 (.61-.66)	.63 (.60-.65)	.64 (.61-.66)	.65 (.62-.68)
7. I’ve been thinking clearly	.59 (.57-.62)	.60 (.57-.62)	.61 (.58-.63)	.62 (.59-.65)	.63 (.60-.66)	.64 (.61-.67)
8.I’ve been feeling good about myself	.65 (.62-.67)	.62 (.60-.65)	.64 (.62-.66)			
9.I’ve been feeling close to other people	.58 (.55-.60)	.58 (.56-.61)	.58 (.56-.61)	.53 (.50-.56)	.54 (.51-.57)	.54 (.51-.57)
10.I’ve been feeling confident	.67 (.65-.70)	.68 (.66-.70)	.69 (.67-.71)			
11.I’ve been able to make up my own mind about things	.66 (.64-.68)	.66 (.64-.69)	.68 (.65-.70)	.62 (.59-.65)	.63 (.61-.66)	.65 (.62-.68)
12.I’ve been feeling loved	.58 (.55-.60)	.58 (.56-.61)	.60 (.57-.62)			
13.I’ve been interested in new things	.49 (.47-.52)	.48 (.45-.51)	.48 (.45-.51)			
14.I’ve been feeling cheerful	.59 (.56-.61)	.56 (.54-.59)	.57 (.54-.60)			

Models with collapsed categories presented negligibly better fit indices than the correlated-residuals models, and practically identical reliability coefficients or factorial loadings (maximum difference < 0.02 for either loadings or reliability).

#### Other instruments’ CFA

The same rationale was applied to the other instruments we included in the survey questionnaire for the purposes of validation. WHO-5 did not present any significantly misspecified parameter. ONS-4 presented severely misspecified parameters, though releasing constraints was not feasible due to the low number of items and, therefore, lack of degrees of freedom.

Only WHO-5's unit-weighted composite scores were acceptable and adequate for general purposes such as research (ω ≥ 0.70); though not for high-stakes decisions (ω < 0.90). On the other hand, the factor scores of all but ONS-4 presented acceptable (FD ≥ 0.80, H ≥ 0.70) to good (FD ≥ 0.90, H ≥ 0.85) determinacy and replicability. GHQ-12 offered an interesting example of the relevance of accounting for factorial structure when assessing the reliability of scores. Though α (not informed by factorial structure) is acceptable (≥ 0.70) or good (≥ 0.90) and the same disregarding the presence of method factors, ω is only acceptable in the severely misspecified unidimensional model and one of the three-correlated factors. In the models with method factors accounting for method-implied correlated residuals, ω is either questionable (< 0.70) or poor (< 0.50). This implies that GHQ-12's unit-weighted composite scores appeared unsuited for practical use.

#### Measurement invariance

Both WEMWBS and SWEMWBS met all criteria for strong measurement invariance (ΔCFI ≥ -0.01, ΔRMSEA ≤ 0.01, ΔSRMR ≤ 0.015) across gender, rural/urban districts, and age groups (Table 5, only WEMWBS shown). All instruments were invariant across rural/urban districts. WHO-5 also presented strong invariance across age and gender. ONS4 showed strong invariance across gender, and partial invariance across age groups (releasing intercept of item 2). GHQ-12 was partially invariant across age (released intercept of item 8) and gender (released intercepts of items 3, 4, 5, and 9).

**Table 5:** WEMWBS’s measurement invariance across sex, rural/urban districts, and age group

	χ^2^	*df*	CFI	RMSEA	SRMR	∆χ^2^	∆*df*	p-value	∆CFI	∆RMSEA	∆SRMR
**Stage (student vs adult sample)**											
Configural	725	148	.949	.065	.044						
Thresholds	930	172	.982	.035	.044	19.1	24	.7455	.0338	-.0297	.0000
Loadings (weak, metric)	1108	185	.987	.029	.046	9.9	13	.7043	.0049	-.0063	.0026
Intercepts (strong, scalar)	1600	198	.981	.034	.048	132.1	13	< .0001	-.0065	.0054	.0016
**Sex**											
Configural	737	148	.954	.062	.044						
Thresholds	835	172	.959	.054	.044	57.0	24	.0001	.0051	-.0077	.0000
Loadings (weak, metric)	901	185	.968	.046	.045	15.0	13	.3279	.0091	-.0080	.0013
Intercepts (strong, scalar)	1032	198	.967	.045	.046	49.0	13	< .0001	-.0010	-.0009	.0010
**Rural/urban**											
Configural	756	148	.955	.061	.044						
Thresholds	808	172	.976	.042	.044	10.8	24	.9901	.0206	-.0195	.0000
Loadings (weak, metric)	848	185	.984	.033	.045	4.2	13	.9886	.0079	-.0087	.0008
Intercepts (strong, scalar)	950	198	.983	.033	.046	36.3	13	.0003	-.0012	.0002	.0007

We also compared the reliability of the scores by group. Both unit-weighted composites and factor scores for the adult group were more reliable for the WEMWBS (ω = 0.79 *vs* 0.71; H = 0.92 *vs* 0.88), SWEMWBS (ω = 0.78 *vs* 0.69; H = 0.85 *vs* 0.78), and WHO-5 (ω = 0.80 *vs* 0.73; H = 0.88 *vs* 0.80) than for the other instruments examined. In conjunction with the analysis of measurement equivalence through CFA, this means that scores in the teenager population carry more "noise", despite measuring the same construct with substantively equal meaning given a specific value. Difference in reliability across gender or rural/urban districts were ≤ 0.02 in either ω, H, or FD coefficients.

#### Criterion validity

Variables correlated as expected, though with weaker coefficients than anticipated (Table 6). When analysing these correlations within groups, we observed that the correlations in the adults sub-sample were closer to the expected values based on previous studies, while in the students' sample some correlations were significantly weaker. For example, in adults WEMWBS and SWEMWBS presented higher correlations with WHO-5 (Δρ = 0.11 and 0.15 respectively), ONS4 (Δρ = 0.11 and 0.13), and self-reported health (Δρ = 0.19 and 0.22).

**Table 6:** Correlation of (S)WEMWBS scores with scores of related instruments and their differences between students and adults

Age group	WHO5	ONS4	GHQ12	WEMWBS	SWEMWBS
**Whole Sample**					
WEMWBS	.54 (.52-.57)	.31 (.28-.35)	.39 (.36-.43)		
SWEMWEBS	.49 (.46-.52)	.28 (.25-.32)	.36 (.33-.39)	.93 (.92-.93)	
Health				.24 (.20-.28)	.20 (.16-.24)
**Students**					
WEMWBS	.52 (.49-.56)	.29 (.25-.33)	.38 (.34-.42)		
SWEMWBS	.47 (.43-.50)	.26 (.22-.30)	.34 (.30-.38)	.92 (.92-.93)	
Health				.21 (.16-.25)	.16 (.11-.20)
**Adults**					
WEMWBS	.64 (.58-.69)	.40 (.33-.48)	.44 (.37-.51)		
SWEMWBS	.61 (.55-.67)	.39 (.32-.47)	.43 (.36-.51)	.95 (.94-.96)	
Health				.40 (.29-.50)	.37 (.27-.48)
**∆Adults-Students**					
WEMWBS	.11 (.05, .18)****	.11 (.03, .20)***	.05 (-.02, .13)		
SWEMWBS	.15 (.08, .21)****	.13 (.05, .22)****	.09 (.01, .17)**	.02 (.01, .03)****	
Health				.19 (.08, .30)****	.22 (.10, .33)****

The difference in the correlation coefficients was modelled through multigroup path analysis using unit-weighted composite scores in an SEM framework. The difference was included as a defined parameter within the model, and therefore it's confidence interval and significance based on the standard error of such parameter

No significant differences in correlation coefficients were observed across gender or rural/urban districts.

### Secondary aim: Distribution and factors associated with mental wellbeing

In the student sample, the mean WEMWBS score was 53.5, with a standard deviation of 9.04. Univariable models found that being male and living in an urban area were associated with better mental wellbeing, the odds of low wellbeing decreased with age (with no corresponding increase in the odds of high wellbeing with age). Adolescents in higher forms (more years of education) had better mental wellbeing than those in lower forms. Adolescents with better self-reported health had better wellbeing that those with poor self-reported health. As expected those classified as socially ‘marginalised’ had poor mental wellbeing compared with those who were not marginalised and the more marginality indicators that an adolescent indicated as applying to them, the poorer their mental wellbeing was likely to be. Those selecting the third option “other” as their gender appear to have the poorest mental wellbeing (Table 7).

**Table 7:** Univariable associations between mental wellbeing (WEMWBS) and individual characteristics in the student sample

Student Survey Participants											
	***n* (%)**	**Mean**	**S.d**	**LQT (*n*)**	**LQT (%)**	**Odds Ratio of LQT score**	***P*-value chi-2**	**UQT (*n*)**	**UQT (%)**	**Odds Ratio of UQT score**	***P*-value chi2**
**Sex**											
Female	1588 (52.03)	52.35	9.06	482	30.4	**ref**	0.000	378	23.8	**ref**	0.000
Male	1458 (47.77)	54.65	8.88	295	20.2	0.58 (0.49, 0.69)		464	31.8	1.49 (1.27, 1.75)	
Other	6 (0.20)	51.00	5.93	2	33.3	1.15 (0.21, 6.28)		0	0	No data	
**Age group**											
15 years	1099 (36.01)	53.07	9.28	310	28.2	**ref**	0.03	287	26.1	**ref**	0.51
16 years	881 (28.87)	53.21	8.88	231	26.2	0.90 (0.74, 1.10)		242	27.5	1.07 (0.88, 1.31)	
17 years	681 (22.31)	53.78	8.92	150	22.0	0.72 (0.57, 0.90)		196	28.8	1.14 (0.92, 1.42)	
18 years	291 (9.53)	54.38	9.09	67	23.0	0.76 (0.56, 1.03)		90	30.9	1.27 (0.95, 1.68)	
19 + years	100 (3.28)	54.76	8.25	21	21.0	0.68 (0.41, 1.11)		27	27.0	1.05 (0.66, 1.66)	
**Form**											
Form one	759 (24.87)	53.06	9.54	216	28.5	**ref**	0.01	198	26.1	**ref**	0.01
Form two	974 (31.91)	52.68	8.91	267	27.4	0.95 (0.77, 1.17)		238	24.4	0.92 (0.74, 1.14)	
Form three	834 (27.33)	54.19	8.87	186	22.3	0.72 (0.58, 0.91)		262	31.4	1.30 (1.04, 1.61)	
Form four	485 (15.89)	54.32	8.64	110	22.7	0.74 (0.57, 0.96)		144	29.7	1.20 (0.93, 1.54)	
**Location**											
Rural	2374 (77.79)	53.00	9.21	651	27.4	**ref**	0.000	628	26.5	**ref**	0.01
Urban	678 (22.21)	55.01	8.26	128	18.9	0.62 (0.50, 0.76)		214	31.6	1.28 (1.06, 1.54)	
**Marginality Indicator**											
Not Marginalized	210 (6.88)	56.96	8.43	28	13.3	**ref**	0.000	93	44.3	**ref**	0.000
Marginalized	2842 (93.12)	53.19	9.04	751	26.4	2.33 (1.55, 3.51)		749	26.4	0.45 (0.34, 0.60)	
**Marginality Score**											
0	210 (6.88)	56.96	8.43	28	13.3	**ref**	0.000	93	44.3	**ref**	0.000
1	363 (11.89)	56.27	8.40	58	16.0	1.24 (0.76, 2.01)		138	38.0	0.77 (0.55, 1.09)	
2	394 (12.91)	54.68	9.40	81	20.6	1.68 (1.05, 2.68)		132	33.5	0.63 (0.45, 0.89)	
3	429 (14.06)	53.88	8.76	96	22.4	1.88 (1.19, 2.96)		123	28.7	0.51 (0.36, 0.71)	
4	348 (11.40)	53.63	9.38	91	26.2	2.30 (1.45, 3.66)		98	28.2	0.49 (0.34, 0.71)	
5	332 (10.88)	52.23	8.49	99	29.8	2.76 (1.74, 4.38)		70	21.1	0.34 (0.23, 0.49)	
6	302 (9.90)	52.16	8.40	81	26.8	2.38 (1.49, 3.82)		58	19.2	0.30 (0.20, 0.44)	
7	219 (7.18)	51.67	8.64	73	33.3	3.25 (2.00, 5.29)		46	21.0	0.33 (0.22, 0.51)	
8	184 (6.03)	50.77	8.94	66	35.9	3.64 (2.21, 5.99)		33	17.9	0.27 (0.17, 0.44)	
9	99 (3.24)	51.46	9.00	31	31.3	2.96 (1.66, 5.30)		22	22.2	0.36 (0.21, 0.62)	
10	83 (2.72)	50.64	9.75	35	42.2	4.74 (2.63, 8.55)		17	20.5	0.32 (0.18, 0.59)	
11 +	89 (2.92)	49.10	9.13	40	44.9	5.31 (2.98, 9.45)		12	13.5	0.20 (0.10, 0.38)	
**Self-Reported Health**											
Excellent	683 (22.38)	55.85	9.45	122	17.9	**ref**	0.000	268	39.2	**ref**	0.000
Very good	247 (8.09)	55.11	8.61	48	19.4	1.11 (0.77, 1.61)		83	33.6	0.78 (0.58, 1.06)	
Good	1250 (40.96)	53.41	8.58	301	24.1	1.46 (1.15, 1.84)		319	25.5	0.53 (0.43, 0.65)	
Fair	835 (27.36)	51.21	8.85	289	34.6	2.43 (1.91, 3.10)		165	19.8	0.38 (0.30, 0.48)	
Poor	37 (1.21)	49.76	10.46	19	51.4	4.85 (2.47, 9.52)		7	18.9	0.36 (0.16, 0.83)	

In the adult sample, the mean WEMWBS score was 55.9 with a standard deviation of 8.34. The only significant association with high mental wellbeing in the univariable analyses identified an association between high self-reported health and high mental wellbeing. There were more significant associations between individual characteristics and low mental wellbeing: increasing age was associated with lower odds of low mental wellbeing; compared with learner guides, teacher mentors and teachers were less likely to have low wellbeing; urban adults are less likely to have low wellbeing than rural adults; more educated adults are less likely to have low wellbeing than less educated adults; and skilled manual workers are more likely to have low wellbeing than teachers (Table 8).

**Table 8:** Univariable associations between mental wellbeing (WEWMBS) and individual characteristics in the adult sample

Adult Survey Participants											
	***n* (%)**	**Mean**	**S.d**	**LQT (*n*)**	**LQT (%)**	**Odds Ratio of LQT score**	***P*-value Chi-2**	**UQT (*n*)**	**UQT (%)**	**Odds Ratio of UQT score**	***p*-value chi-2**
**Sex**											
Female	459 (79.97)	55.47	8.68	135	29.4	**1.00 (Reference)**	0.067	116	25.3	**1.00 (Reference)**	0.190
Male	115 (20.03)	57.74	6.58	24	20.9	0.63 (0.39, 1.04)		36	31.3	1.35 (0.86, 2.11)	
**Age group**											
15–19 years	73 (12.72)	52.41	10.74	29	39.7	**1.00 (Reference)**	0.005	13	17.8	**1.00 (Reference)**	0.517
20–24 years	162 (28.22)	54.47	9.22	56	34.6	0.80 (0.45, 1.42)		42	25.9	1.62 (0.81, 3.24)	
25–29 years	75 (13.07)	56.57	7.60	19	25.3	0.51 (0.26, 1.04)		19	25.3	1.57 (0.71, 3.46)	
30–34 years	160 (27.87)	57.79	6.72	34	21.3	0.41 (0.22, 0.75)		47	29.4	1.92 (0.96, 3.82)	
35–39 years	79 (13.76)	57.47	6.44	14	17.7	0.33 (0.16, 0.69)		24	30.4	2.01 (0.93, 4.34)	
40 + years	25 (4.36)	56.76	6.98	7	28.0	0.59 (0.22, 1.59)		7	28.0	1.79 (0.62, 5.18)	
**Role**											
Learner Guide	304 (52.96)	54.65	9.48	102	33.6	**1.00 (Reference)**	0.004	77	25.3	**1.00 (Reference)**	0.676
Teacher Mentor	90 (15.68)	57.03	6.64	18	20.0	0.50 (0.28, 0.87)		23	25.6	1.01 (0.59, 1.74)	
Teacher	180 (31.36)	57.51	6.54	39	21.7	0.55 (0.36, 0.84)		52	28.9	1.20 (0.79, 1.81)	
**Location**											
Rural	443 (77.18)	55.64	8.45	135	30.5	**1.00 (Reference)**	0.006	111	25.1	**1.00 (Reference)**	0.155
Urban	131 (22.82)	56.88	7.94	24	18.3	0.51 (0.31, 0.83)		41	31.3	1.36 (0.89, 2.09)	
**Education**											
No Higher Education	286 (49.83)	54.38	9.53	99	34.6	1.00 (Reference)	0.000	69	24.1	**1.00 (Reference)**	0.203
Some Higher Education	288 (50.17)	57.45	6.64	60	20.8	0.50 (0.34, 0.72)		24.13	28.8	1.27 (0.88, 1.85)	
**Self-Reported Health**											
Excellent	185 (32.23)	59.74	6.28	18	9.7	**1.00 (Reference)**	0.000	73	39.5	**1.00 (Reference)**	0.000
Very good	87 (15.16)	57.30	7.63	17	19.5	2.25 (1.10, 4.63)		24	27.6	0.58 (0.34, 1.02)	
Good	264 (45.99)	53.58	8.49	105	39.8	6.13 (3.55, 10.57)		49	18.6	0.35 (0.23. 0.54)	
Fair to Poor	38 (6.62)	50.47	9.31	19	50.0	9.28 (4.17, 20.66)		6	15.8	0.29 (0.11, 0.72)	
**Main Occupation**											
Teacher	266 (46.34)	57.20	6.68	60	22.6	**1.00 (Reference)**	0.009	73	27.4	**1.00 (Reference)**	0.765
Professional	26 (4.53)	58.35	7.19	3	11.5	0.45 (0.13, 1.54)		8	30.8	1.18 (0.49, 2.82)	
Skilled Non Manual	100 (17.42)	55.71	8.19	30	30.0	1.47 (0.88, 2.46)		25	25.0	0.88 (0.52, 1.49)	
Skilled Manual	111 (19.34)	53.00	10.12	44	39.6	2.25 (1.40, 3.63)		24	21.6	0.73 (0.43, 1.23)	
Unskilled	10 (1.74)	56.30	9.36	3	30.0	1.47 (0.37, 5.86)		3	30.0	1.13 (0.29, 4.50)	
Other	61 (10.63)	54.90	10.38	19	31.2	1.55 (0.84, 2.87)		19	31.2	1.20 (0.65, 2.19)	

In the student multivariable model, males have 0.6 (95% CI 0.51–0.72) times the odds of low wellbeing and 1.41 (95% CI 1.20–1.66) times the odds of high wellbeing compared to females. The number of students selecting “other” for their sex were too small in number to draw firm conclusions. Students in form 3 and 4 had significantly lower odds of low mental wellbeing that students in form 1 do (0.63 (95% CI 0.50–0.80) and 0.75 (95% CI 0.57–0.99) respectively). Students in form 3 also had significantly higher odds of high mental wellbeing compared with students in form 1 (1.46 (95% CI 1.20–1.66)). Urban students had lower odds of low mental wellbeing and higher odds of high mental wellbeing compared with rural students (0.61 (95% CI 0.49–0.76), 1.25 (95% CI 1.03–1.53) respectively). Students categorised as marginalised had higher odds of low mental wellbeing and lower odds of high mental wellbeing (1.76 (1.16–2.67) and 0.56 (0.41–0.75) respectively). Finally, there were increasing odds of poor mental wellbeing and decreasing odds of high mental wellbeing for those self-reporting poorer health (Table 9).

**Table 9:** Student sample multivariable model results

Variable	Categories	Adjusted Odds Ratio of LQT score (95% Confidence Interval)	Adjusted Odds Ratio of UQT score (95% Confidence Interval)
Sex	Female	**1.00 (Reference)**	**1.00 (Reference)**
	Male	0.60 (0.51, 0.72)	1.41 (1.20, 1.66)
	Other	1.11 (0.20, 6.11)	! Predicts failure perfectly
Form	Form one	**1.00 (Reference)**	**1.00 (Reference)**
	Form two	0.95 (0.77, 1.19)	0.92 (0.74, 1.16)
	Form three	0.63 (0.50, 0.80)	1.46 (1.17, 1.83)
	Form four	0.75 (0.57, 0.99)	1.27 (0.97, 1.66)
Urban/Rural	Rural	**1.00 (Reference)**	**1.00 (Reference)**
	Urban	0.61 (0.49, 0.76)	1.25 (1.03, 1.53)
Marginality	Not Marginalized	**1.00 (Reference)**	**1.00 (Reference)**
	Marginalized	1.76 (1.16, 2.67)	0.56 (0.41, 0.75)
Self-Reported Health	Excellent	**1.00 (Reference)**	**1.00 (Reference)**
	Very good	1.11 (0.76, 1.61)	0.77 (0.57, 1.05)
	Good	1.42 (1.12, 1.81)	0.54 (0.57, 0.66)
	Fair	2.24 (1.82, 3.00)	0.40 (0.32, 0.51)
	Poor	4.94 (2.48, 9.85)	0.37 (0.16, 0.85)

In the adult multivariable model, the only significant associations were for location (with urban adults less likely to have low mental wellbeing OR 0.51 (0.31–0.83)) and for self-reported health with both low and high mental wellbeing (Table 10).

**Table 10:** Adult multivariable analysis

Variable	Categories	Adjusted Odds Ratio of LQT score (95% Confidence Interval)	Adjusted Odds Ratio of UQT score (95% Confidence Interval)
Sex	Female	**1.00 (Reference)**	**1.00 (Reference)**
	Male	1.02 (0.51, 2.04)	1.24 (0.67, 2.30)
Age group (years)	15–19 years	**1.00 (Reference)**	**1.00 (Reference)**
	20–24 years	0.98 (0.52, 1.83)	1.45 (0.71, 2.97)
	25–29 years	0.58 (0.26, 1.30)	1.67 (0.72, 3.90)
	30–34 years	0.58 (0.22, 1.56)	2.57 (0.95, 6.96)
	35–39 years	0.48 (0.15, 1.47)	2.67 (0.89, 7.97)
	40 + years	0.93 (0.24, 3.62)	2.41 (0.63, 9.19)
Role	Learner Guide	**1.00 (Reference)**	**1.00 (Reference)**
	Teacher Mentor	0.55 (0.14, 2.22)	0.50 (0.16, 1.56)
	Teacher	0.66 (0.17, 2.52)	0.52 (0.17, 1.59)
Urban/Rural	Rural	**1.00 (Reference)**	**1.00 (Reference)**
	Urban	0.51 (0.31, 0.83)	1.33 (0.84, 2.10)
Education	Did not attend Higher Education	**1.00 (Reference)**	**1.00 (Reference)**
	Attended Higher Education	1.11 (0.36, 3.48)	1.25 (0.50, 3.12)
Self-Reported Health	Excellent	**1.00 (Reference)**	**1.00 (Reference)**
	Very good	2.46 (1.17, 5.14)	0.57 (0.32, 1.00)
	Good	6.56 (3.72, 11.54)	0.37 (0.24, 0.57)
	Fair to Poor	9.53 (4.11, 22.11)	0.32 (0.12, 0.81)

## Discussion

Overall, with respect to our primary aim: the Swahili translation of WEMWBS and its short form were applicable, understood, and relevant to GEC participants which was demonstrated through the qualitative data and through the 100% completion rate of the survey. Additionally, they met quantitative tests of reliability and validity i.e.: they were correlated with comparator scales and met the criteria to determine a single factor structure. This Swahili translation of WEMWBS is now available for use (https://warwick.ac.uk/fac/sci/med/research/platform/wemwbs/using/translations/). With respect to the secondary aim of the study: for students in the GEC supported government schools mental wellbeing is higher in students in the final two ‘forms’ of school compared with the first two. In addition, being male, urban residence, the absence of markers of marginality and better self-reported health were all significantly associated with better mental wellbeing. For adults, urban residence and better self-reported health were associated with better mental wellbeing.

In our study both unit-weighted composites and factor scores for the adult group were more reliable for the WEMWBS, SWEMWBS, and WHO-5 than for ONS-4 and GHQ-12, this implies that for this adult sample the reliability of (S)WEMWBS and WHO-5 is distinctively higher than that of the other instruments we collected. WHO-5’s scores were the only ones with acceptable reliability (omega > 0.70) besides (S)WEMWBS. On the one hand, this makes the correlation between (S)WEMWBS and WHO-5 scores the most interesting for assessing criterion validity, since the other scores are not as reliable. On the other hand, it seems sensible to recommend the use of either version of (S)WEMWBS in further wellbeing studies when there is an interest in observing mental wellbeing specifically, and not physical wellbeing – since WHO-5 conflates both into a general wellbeing measurement. If such general wellbeing were the exclusive interest, WHO-5 might produce more reliable scores than ONS-4. Although WEMWBS and GHQ-12 have slightly different foci – mental wellbeing vs ill-health, the unsatisfactory reliability of the observed GHQ-12 scores in the population studied in this validation, might imply that WEMWBS is a better option for studying mental health in normal populations in Tanzania. This would be emphasized if the length of the questionnaire was of concern, since SWEMWBS offers an economical alternative with no loss of reliability when the intended use is unit-weighted composite scores. Of course, these observations on the adequacy of structural models and the reliability of scores of each instrument need to be further studied in other Swahili-speaking populations.

It was interesting that one aspect of mental wellbeing alluded to by focus group participants was the contribution of individual wellbeing to community wellbeing. Those with higher wellbeing were thought to make a positive contribution to the wider community. This attribute of mental wellbeing is not captured by WEMWBS other than through the item ‘I feel useful’ or by any of the other tools included in our study. Amongst other available wellbeing measures, the mental health continuum short form may have items that captured this element of mental wellbeing and could be further explored for use in Tanzania. For example in the mental health continuum question “During the past month, how often have you felt the following?”, Items include: “Part of your community”, “important to your society” [17].

Average scores in the population of this study were higher than in other populations [18–20] and there was a tendency for sparse data in the lowest response categories for some items, and particularly among men. This general uplift in average score may be related to some stigmatizing attitudes towards poor mental health expressed in the focus groups, and an increased likelihood of social desirability bias. WEMWBS (as a self-completion questionnaire) might partially mitigate against social desirability bias, and because it offers a positively framed approach to mental health, that may be more acceptable to this population. Despite this, it is possible that mean population norms from this population are inflated and that could have implications when comparing with other populations. Men may have better wellbeing, or may have greater score inflation, perhaps because gender norms influence the degree of social desirability bias. There is evidence of this from other cultural contexts [21], and this could be explored further. Measurement invariance analysis would be valuable to mitigate this possible effect when comparing different populations' scores.

In terms of the epidemiology of mental wellbeing in our GEC population, one of the striking findings in adolescents is the improvement in mental wellbeing in Form 3. Students sit exams at the end of their Form 2 year, and it may be that exam stress undermines wellbeing as adolescents approach these exams, or there may be survivor bias (the students who ‘survive’ the exam and continue with their education in Form 3 are the most resilient). Better wellbeing among male students compared with female students, and compared with those who did not wish to indicate their sex (although those numbers were small) is in line with literature that suggests greater rates of minor mental illness among females compared with males [22] and poor wellbeing among transgender people who often face stigma, discrimination and exclusion[23]. Better wellbeing among those with fewer marginality indicators was also expected, as these marginality indicators all suggest disadvantage for the students. The well-evidenced bidirectional link between physical and mental health is likely responsible for the association between better wellbeing and better self-reported health[24]. The urban advantage is likely due to better access to resources and opportunities in urban areas. It was unexpected that there was no gender difference in mental wellbeing for adults, we hypothesise that this is because our adult sample is not representative and may include particularly resilient women (secondary school completers and teachers).

### Strengths and limitations

This was a well-powered study, using Gold standard translation to develop a version of WEMWBS in Swahili. The methodological decisions for CFA aimed for an analysis that meets the literature standards better than often seen in similar practical applications. This allowed a more rigorous and informative assessment of the strengths and limitations of WEMWBS. For example, examining the data as ordinal with an adequate estimator allowed us to examine the implications of sparse data in the lowest (worst) response categories – thereby tackling a plausible limitation of the data and potential explanations for general tendencies, like the high scores when contrasted with previous international studies.

Another example of the high-quality quantitative analysis was the use of omega instead of alpha, which allowed us to observe that the scores of both WEMWBS and SWEMWBS are as reliable (0.71 and 0.70 respectively) for using unit-weighted composites, while an assessment based on alpha would have indicated that WEMWBS gave more reliable scores (0.88 vs 0.79). We further took advantage of this through the systematic evaluation and reporting of the correlated residuals included in the models, alongside their effects – or lack thereof – on the substantive meaning of the scores, since these correlations were considered by omega, and further contrasted examining their RD with the theoretical model’s loadings. Reporting H and FD also informed us that, although the Swahili versions of both SWEMWBS and WEMWBS’ scores are acceptable for calculating factor scores or modelling measuring models in an SEM framework, the full WEMWBS is better for these purposes. This information might guide decisions on which version to use depending on the resources available and the goals of the implementation. We can observe advantages also in the use of CFA to assess the scores of other instruments intended for examining criterion validity and further practical recommendations for measuring wellbeing in the target population.

By using a mixed methods approach we were able to examine both the acceptability of the translated (S)WEMWBS for measuring mental wellbeing for researchers and for participants. This also allowed us to examine whether qualitative participants discussed anything challenging or unexpected about the items that quantitative analyses picked up as less consistent (for example WEMWBS items 4 and 13, which in this case was reassuring).

It is worth noting that neither student nor adult sample are representative of the general population in Tanzania: the student sample is particularly deprived- from schools identified for extra support through the Girls Education Challenge fund. Meanwhile the adult sample is dominated by teachers and likely to be privileged compared to an adult sample from the general population. We completed data collection in August 2020, during the COVID-19 pandemic. Students had recently returned to schools but everyone was in the midst of change and uncertainty. This means it is unlikely that we can generalise the findings from this study to students and adults in Tanzania in general.

## Conclusions

Swahili WEMWBS has been validated for use in populations aged 15 + years. It can now be accessed and used to measure mental wellbeing in relevant groups. When making international comparisons, it is important to consider measurement invariance analysis, given evidence presented here of higher mean scores that may be routed in social desirability bias.

Our analysis is being used by CAMFED to develop their life skills programme and teacher training contents, in order to improve mental wellbeing throughout the GEC funded schools to promote better educational outcomes for their students.

## Supplementary Information

**Additional file 1.**

**Additional file 2.**

## Data Availability

The datasets generated and analysed during the current study are available from the corresponding author on reasonable request.

## Abbreviations

CAMFED: Campaign for female education
CFA: Confirmatory Factor Analysis
CFI: Comparative Fit Index
GEC: Girls Education Challenge
GHQ-12: The 12-item General Health Questionnaire (GHQ-12)
ONS-4: Office for National Statistics-4
RD: Relative Difference
RMSEA: Root Mean Square Error of Approximation
SRMR: Standardized Root Mean Square Residual
SWEMWBS: Short Warwick Edinburgh Mental Wellbeing Scale
TLI: Tucker-Lewis Index
WEMWBS: Warwick Edinburgh Mental Wellbeing Scale
WHO-5: The World Health Organisation- Five Well-being Index
