# Supplementary material for: Swahili translation and validation of the Warwick Edinburgh Mental Wellbeing Scale (WEMWBS) in adolescents and adults taking part in the girls’ education challenge fund project in Tanzania
Source: Health Qual Life Outcomes. 2023 May 10;21:43. doi: 10.1186/s12955-023-02119-9 (PMC10171168; doi:10.1186/s12955-023-02119-9)
Supplement: Supplementary file 1 — Additional file 1. [file 12955_2023_2119_MOESM1_ESM.docx]

**Understanding wellbeing in the Girls’ Education Challenge Fund project in Tanzania: Focus Group Schedule**

- Introduction
- Participant information
- Discussion rules
- Assent to record

Some people say that good mental wellbeing can mean being comfortable, happy, healthy and satisfied. Some people think that mental wellbeing includes how satisfied we are with our lives, and our day-to-day emotions (happiness and anxiety)

- What does wellbeing mean to you?
  - What does positive wellbeing mean to you? How would you feel if you have great wellbeing?
  - How does a person feel when they have poor wellbeing?

Earlier/yesterday you answered a series of questions that were trying to measure your wellbeing.

- Which questions can you remember?

Here are the questions (hand-outs). Let’s look at WEMWBS first.

- Were any of these difficult to understand?
- Were any of them difficult to answer?

(Go through each item to prompt further reflection)

- Did you feel able to answer truthfully?
- How do you feel about these questions?
- Do you think someone who looks at your answers to these questions will get a good picture of your wellbeing overall, or is anything missing?

Let’s look at the other questions too (SF-6D, WHO-5, GHQ-12).

- How do these different questions compare?
  1. Which was your favourite set of questions to answer or which were the least annoying/difficult set of questions to answer?
  2. Do any of these best capture what you think of when you think of wellbeing?
- Is there anything else you would like to say about your experience completing these questions?
- Is there anything else you would like to say about what you think wellbeing means?
