# Supplementary material for: Swahili translation and validation of the Warwick Edinburgh Mental Wellbeing Scale (WEMWBS) in adolescents and adults taking part in the girls’ education challenge fund project in Tanzania
Source: Health Qual Life Outcomes. 2023 May 10;21:43. doi: 10.1186/s12955-023-02119-9 (PMC10171168; doi:10.1186/s12955-023-02119-9)
Supplement: Supplementary file 2 — Additional file 2. [file 12955_2023_2119_MOESM2_ESM.docx]

**Supplementary materials 1: Detailed quantitative methods for validation study.**

**Confirmatory factor analysis**

Confirmatory factor analysis (CFA) was performed in three steps: (1) specification of theoretical model, (2) modification based on potentially misspecified parameters, and (3) assessment of global fit of the modified model and the relative difference (RD) of parameters between theoretical and the modified model ($\frac{\theta_{modified}-\theta_{theoretical}}{\theta_{modified}}$). The modified model was considered appropriate when (i) no parameters were severely misspecified, (ii) RD in loadings compared to the theoretical model was negligible (max. individual RD < 10%), and (iii) global fit indexes fell within pre-defined ranges of acceptability. This three-step CFA allowed us to model sources of error that could be substantively irrelevant (i.e., produced negligible parameter bias or RD) but improved the precision of reliability indices (diminishing the risk of overestimating scores' reliability).

Models were fitted using unweighted least squares estimator with mean and variance adjusted (scale-shifted approach, ULSMV). This estimator has been shown to yield more precise parameter and standard error estimates than WLSMV (e.g., Forero et al., 2009; Li, 2016), especially when using sparse data (DiStefano et al., 2020; Savalei & Rhemtulla, 2013), as is the case for this study (see details below).

**Specification of the models.** Models were specified with no marker indicator and standardizing the latent variable (sd = 1, mean = 0).

**Misspecification and modification**. Following Saris, Satorra and van der Veld (2009), the potential misspecification of the models was explored using expected parameter change (EPC) in combination with the modification index (MI) and the power of the MI test. This was independent of the models' global fit, which can both under- and over-estimate misspecified parameters. This allowed us to examine where the model was potentially misspecified and whether such misspecification was trivial or severe. A parameter was considered misspecified when (a) the MI was significant and power low or (b) the MI was significant, the power high, and the EPC large (i.e., greater than the target EPC for each parameter as suggested by Saris et al., 2009, p. 571). Then, the misspecification was considered significant when the confidence interval of the EPC fell outside the range of trivial misspecification. Under this condition, if the misspecified parameter was likely implied by measurement (e.g., wording effects; common themes), the model was refitted, freeing the parameter, and its substantive interpretation was discussed.

**Global fit and parameter relative difference (RD)**. Once potential areas of misspecification were analyzed, the global fit of the theoretical and modified models was assessed. Given the increased sensitivity of χ^2^ to slight misspecification with large samples, several alternative fit indices where used. Comparative Fit index (CFI) and Tucker-Lewis Index (TLI) were considered acceptable over .90, and good when close to or over .95. Root Mean Square Error of Approximation (RMSEA) and Standardized Root Mean Square Residual (SRMR) were considered acceptable when close to or under .08 and good when close to or under .06. Finally, we analysed the relative difference or bias in the factorial loads that such respecification produced. Using cutoffs from prior research (e.g. DiStefano et al., 2020; Muthén et al., 1987), maximum individual RDs with absolute values less than 10% were considered acceptable.

**Internal consistency, determinacy and replicability.** Once a modified model was retained, reliability indices for scores were analysed. For assessing the internal consistency of unit-weighted composite scores (sum or mean of observed scores), MacDonald's ω was chosen instead of Cronbach's α since the former accounts for factor structure (including, for example, correlated residuals) and is more appropriate when the loadings vary – α is reported for discussion. Two sets of cut-off values have been considered for ω. One is a practically oriented pair in which values > .70 are adequate for general purposes such as research whereas values > .90 are expected for high-stakes decisions. Besides this, a traditional, scaled rule-of-thumb has also been considered: excellent ( > .90), good (>.80), acceptable (>.70), questionable (>.60) and poor (>.50). Alongside reporting ω to assess whether unit-weighted scoring was justifiable, we evaluated whether calculating factor scores for further analysis or specifying measurement models in an SEM framework was reasonable. For this assessment, we report coefficients of factor score determinacy (FD: acceptable > .80, good >.90 – Grice, 2001) and construct replicability (H: acceptable >.70, good >.85 – Hancock & Mueller, 2001).

**Nonnormality, ceiling effects and collapsing categories**

The data for indicators in all instruments was skewed due to increased concentration in the higher (better) values; with endorsement of the highest response category over 40% in some items. All instruments presented sparse data with more than half of their items having < .5% of endorsement in the lowest (i.e. worse scoring) response category. Therefore, following DiStefano et al. 2020, guidelines, we evaluated each model both with and without collapsed response categories.

**Measurement Equivalence**

Following Wu and Estabrook (2016), measurement invariance for models with ordinal indicators (such as WEMWBS) were analysed using theta parameterization and, after establishing configural invariance, constraining first thresholds to be equal before proceeding to constrain loadings and intercepts to establish weak/metric and strong/scalar invariance. Since χ2 is overly sensitive to small, unimportant deviations from a 'perfect' model in large samples (e.g. Putnick & Bornstein, 2016), we assessed measurement invariance focusing on several fit indices. Following Rutkowski and Svetina (2014) changes in CFI of up to -.02 and RMSEA of up to .03 were considered appropriate for tests of metric/weak invariance, while ΔCFI ≥ -.01 for and ΔRMSEA ≤ .01 were considered appropriate for scalar/strong invariance tests. We also considered Chen's (2007) recommendation of a change in SRMR ≤.030 (for metric invariance) or ≤.015 (for scalar or residual invariance).

**References:**

Chen FF. Sensitivity of Goodness of Fit Indexes to Lack of Measurement Invariance. https://doi.org/101080/10705510701301834. 2007;14(3):464–504.

DiStefano C, Shi D, Morgan GB. Collapsing Categories is Often More Advantageous than Modeling Sparse Data: Investigations in the CFA Framework. https://doi.org/101080/1070551120201803073. 2020;28(2):237–49.

Forero CG, Maydeu-Olivares A, Gallardo-Pujol D. Factor Analysis with Ordinal Indicators: A Monte Carlo Study Comparing DWLS and ULS Estimation. http://dx.doi.org/101080/10705510903203573. 2009;16(4):625–41.

Grice JW. Computing and evaluating factor scores. Psychol Methods. 2001;6(4):430–50.

Hancock G., Mueller R. Rethinking construct reliability within latent variable systems. In: Cudeck R, Jöreskog KG, Sörbom D, Du Toit S, editors. Structural equation modeling: Present and future – a festschrift in honor of Karl Joreskog. Scientific Software International; 2001. p. 195–216.

Li C. Confirmatory factor analysis with ordinal data: Comparing robust maximum likelihood and diagonally weighted least squares. Behav Res Methods . 2015;48(3):936–49.

Muthén B, Kaplan D, Hollis M. On structural equation modeling with data that are not missing completely at random. Psychom . 1987;52(3):431–62.

Putnick DL, Bornstein MH. Measurement invariance conventions and reporting: The state of the art and future directions for psychological research. Dev Rev. 2016;41:71–90.

Rutkowski L, Svetina D. Assessing the Hypothesis of Measurement Invariance in the Context of Large-Scale International Surveys. http://dx.doi.org/101177/0013164413498257. 2014;74(1):31–57.

Saris WE, Satorra A, Veld WM van der. Testing Structural Equation Models or Detection of Misspecifications? http://dx.doi.org/101080/10705510903203433. 2009;16(4):561–82.

Savalei V, Rhemtulla M. The performance of robust test statistics with categorical data. Br J Math Stat Psychol. 2013;66(2):201–23.

Wu H, Estabrook R. Identification of Confirmatory Factor Analysis Models of Different Levels of Invariance for Ordered Categorical Outcomes. Psychometrika. 2016;81(4):1014–45.
